# Echocardiography in cardio-oncology: optimising service delivery

**DOI:** 10.1186/s44156-026-00115-5

**Published:** 2026-04-20

**Authors:** Joshua Lushington, Abhinav Kandala, Jose Bingcang, Ana Ferreira, Alex Byrne, Alex Sirker, Sanjeev Bhattacharyya, Jonathan Lambert, Victoria Grandage, Rachael Windsor, Robin Chung, Alison Macklin, J. Malcolm Walker, Arjun K. Ghosh

**Affiliations:** 1 Cardio-Oncology Service, University College London Hospital, University College London Hospitals NHS Foundation Trust, London, UK; 2 Cardio-Oncology Service, Barts Heart Centre, Barts Health NHS Trust, London, UK; 3 Hatter Cardiovascular Institute, University College London, London, UK; 4 Echocardiography Service, University College London Hospital, University College London Hospitals NHS Foundation Trust, London, UK; 5 Haemato-Oncology Service, University College London Hospital, University College London Hospitals NHS Foundation Trust, London, UK; 6 Late Effects Service, University College London Hospital, University College London Hospitals NHS Foundation Trust, London, UK; 7 William Harvey Research Institute, Queen Mary University of London, London, UK

**Keywords:** Cardio-oncology, Echocardiography, Oncology, Cancer

## Abstract

Echocardiography plays a central role in the cardiovascular assessment and surveillance of patients with active cancer or in remission. With the development of specialised cardio-oncology services, the demand for echocardiography in cancer patients continues to grow. Here we explain the nuances of echocardiography in cardio-oncology, and detail how our institution has optimised service delivery to minimise cancer patient healthcare visits, improve time to cardio-toxicity diagnosis and utilising physiologists to lead cancer survivorship clinics.

**Supplementary Information:**

The online version contains supplementary material available at 10.1186/s44156-026-00115-5.

## Introduction and background

Cardio-oncology services are now well established at many centres worldwide [1]. The main goal of this sub-specialty is to allow patients to complete their cancer treatment safely and with minimal disruption, whilst limiting potential cardiovascular complications [2]. Transthoracic echocardiography (echo) plays a significant role in screening and monitoring cardiac function before, during and after cancer treatment, and is truly the “work horse” of cardio-oncology.

There is an extensive list of cancer therapies recognised to cause cardiovascular toxicity, also known as cancer therapy related cardiac dysfunction (CTRCD) (see Table 1) [3–6]. Cancer patients are also more likely to impacted by all the usual cardiac conditions which affect the general population, compounded by shared risk factors [7, 8]. 

**Table 1:** Examples of cancer therapies, which cancers they commonly treat and examples of clinically encountered cardio-toxicities (note this is not an exhaustive list)

Class of Agent	Common Examples	Common Cancer Treated	Example of Cardio-Toxicity Encountered
Anthracyclines	Doxorubicin, Epirubicin	Breast cancer, Haematological malignancy	Left ventricular dysfunction
Anti-HER2	Trastuzumab, pertuzumab	Breast and gastric cancer	Left ventricular dysfunction (?transient)
Fluoropyrimidines	Fluorouracil (5-FU), capecitabine	Gastro-intestinal malignancy	Coronary vasospasm
Immune Checkpoint Inhibitors (ICI)	Pembrolizumab, nivolumab	Melanoma, Lung cancer	Myocarditis
VEGF Inhibitors	Sunitinib, Bevacizumab	Renal Cell Carcinoma, colorectal cancer	Hypertension
Bruton’s TKI	Ibrutinib	Chronic Lymphocytic leukemia	Atrial fibrillation
BCR-ABL TKI	Dasatinib	Chronic Myeloid Leukemia	Pulmonary Hypertension
Alkylating agents	Cyclophosphamide	Haematological malignancy	Left ventricular dysfunction

The most well recognised cardio-toxicity is from the use of anthracyclines in breast cancer, which cause left ventricular (LV) dysfunction in a dose dependent fashion. This can present acutely in the weeks following treatment or more insidiously after years. Breast cancer patients with human epidermal growth factor receptor 2 (HER2) positive disease may also receive targeted therapies, such as Trastuzumab. This has the potential to further affect LV function, particularly when given in combination with anthracyclines. Guidelines suggest that patients receiving HER2 targeted therapies should have a baseline echo, and then an echo every 3 months during treatment (see Table 2) [9, 10]. Evidently patients receiving these chemotherapies require a baseline echo prior to the commencement of their treatment to help anticipate risk, and monitor for changes between cycles. Often this needs to be coordinated promptly to mitigate clinical deterioration and minimise therapy disruption.

**Table 2:** Examples of breast cancer therapies, and the suggested transthoracic echocardiography (echo) surveillance schedule as per the 2022 European Society of Cardiology (ESC) Guidelines on Cardio-Oncology. Low, moderate, high, and very high risk classification is per such guidelines

Breast Cancer Therapy	Baseline Echo	Schedule During Treatment	Completion Echo	Post Treatment
Anthracycline (e.g. doxorubicin)		Considered for low and moderate risk before cycle 4. Before every 2nd cycle for high and very high risk.		3 months post treatment for high and very high risk. 12 months post treatment for all.
Anti-HER2 (e.g. trastuzumab)		3 monthly for all risk levels.		3 months post treatment for high and very high risk. 12 months post treatment for all.

Echocardiography in a cardio-oncology population has certain nuances that distinguish them from other patient groups. Primarily these relate to the frequency of repeat assessments of LV function and reliance on inter-operator image interpretation. The British Society for Echocardiography (BSE) and the British Cardio-Oncology Society (BCOS) released a guideline for echocardiography in patients receiving anthracyclines and/or Trastuzumab in 2021 [11]. An optimal cardio-oncology echocardiogram will include assessments of LV function with 3D techniques, as well as GLS measurements, and thorough diastolic and right ventricular assessments (see Table 3). GLS likely identifies early cardio-toxicity, before LV ejection fraction (EF) overtly drops [12]. For echocardiographic surveillance, it is essential to compare current findings to previous, particularly GLS and LVEF. If there is suboptimal endocardial definition, then echo-contrast agents should be used to maximise accuracy. In subjects where echo windows are poor, cardiac MRI may be required to accurately define the ventricular dimensions and EF

**Table 3:** Transthoracic Echocardiography (echo) parameters required for a complete cardio-oncology echo, with their clinically relevant findings

Transthoracic Echo Parameter	Clinical Significance
GLS	Reduction by ≥ 15% suggests cardio-toxicity. May develop prior to overt reduction in LVEF.
LVEF and LVEDV (ideally 3D)	Reduction by ≥ 10% suggests cardio-toxicity. 3D echo techniques improve reliability.
Diastolic Parameters	Diastolic dysfunction may proceed over systolic dysfunction.
RV size and function (TAPSE / RVS’ / FAC / FWLS)	May decline in cardiotoxicity. May show “McConnell’s sign” in PE.
Peak TRV	Drugs such as Dasatinib can cause PAH.
Pericardial effusion assessment	Multiple cancer treatments or the malignancy itself can cause effusion and tamponade.
Valvular assessment	Chest wall radiotherapy can predispose to calcific valvular disease.
IVC assessment	May be relevant in RVSP and tamponade assessments. Certain malignancy such as RCC can cause “tumour thrombus” in IVC.
Contrast Echo	If the endocardium of ≥ 2 contiguous LV segments is not clearly defined contrast should be used for accurate LVEF.

LVEF = left ventricular ejection fraction, LVEDV = left ventricular end diastolic volume, GLS = global longitudinal strain, RV = right ventricle, TAPSE = tricuspid annular plane systolic excursion, RVS’ = right ventricular S wave velocity, FAC = fractional area change, FWLS = free wall longitudinal strain, PE = pulmonary embolism, TRV = tricuspid regurgitation velocity, PAH = pulmonary arterial hypertension, IVC = inferior vena cava, RVSP = right ventricular systolic pressure, RCC = renal cell carcinoma, LV = left ventricle

**Fig. 1:**
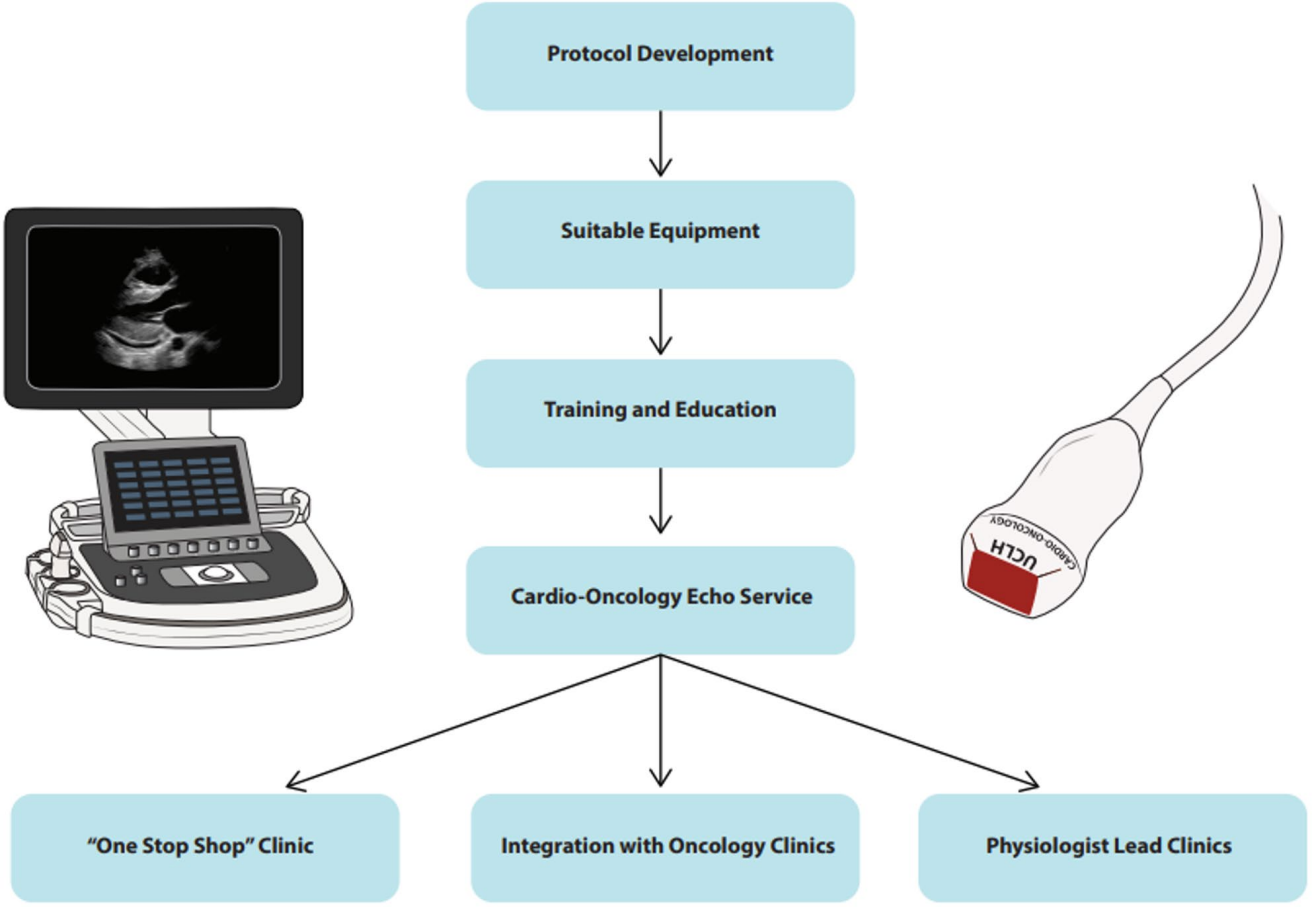
Ongoing evolution of a multi-faceted cardio-oncology echocardiography service

The significant complexity and flexibility required for managing cancer patients mean that echocardiography services in cardio-oncology need to be appropriately designed and positioned to be able to meet the varied demands of this cohort. This in turn offers the prospect for novel approaches in service structure and delivery. The cardio-oncology service at University College London Hospital (UCLH) was established in 2016, and here we detail the novel approaches to cardio-oncology echo service provision (see Fig. 1).

### What did we do? 

### “The one stop shop”

Cancer patients have frequent health care appointments and may spend much of their week in a clinic or hospital [13]. Combined with common side effects of chemotherapy, such as nausea and fatigue, and the cost of transport, leaving the house becomes burdensome and financially stressful. Hence, minimising the time spent attending appointments is of significant benefit. The “one stop shop” refers to facilitating cardio-oncology services to occur on the same day to minimise the number of visits the patient is required to make. In the beginning, the cardio-oncologist would perform the required echocardiogram at the start of the appointment, before proceeding to the usual clinic assessment. Over the last 8 years, the demand for cardio-oncology input has grown dramatically, and the cardio-oncologist no longer has capacity to perform the echocardiograms themselves. To accommodate, a small cohort of engaged and motivated physiologists were recruited and trained in all aspects of cardio-oncology echocardiography. The initial cohort was kept small to ensure optimal quality control and necessary mentorship. An abbreviated echocardiography template was devised for selected surveillance scans, as many patients required “focussed LV assessments.” This template was the precursor to the “targeted” echo approach introduced in the BSE-BCOS cardio-oncology guideline in 2021 [5]. While the number of echocardiographers doing the one-stop clinic list has since expanded, quality control and governance is maintained by a senior cardiac physiologist who has also completed a higher degree in cardio-oncology alongside the cardio-oncologists.

This team of highly trained physiologists conduct scans and prepare reports for patients on the same day as their clinic appointment. The cardio-oncologist then has an accurate up to date echo to review and base clinical decisions on, and the patient has one less clinical encounter. For this to function well, there needs to be fluidity between the echocardiogram and clinic appointments. A cancer diagnosis may be sudden, and there is usually urgency for commencing treatment. Therefore, the service needs to be able to quickly react to accommodate appointments. Thus, a select number of slots are allocated for urgent patients booking at short notice. However, if they are not filled 48 h before the appointment date, the slot is given to a less urgent case, to minimise waiting times. A well-functioning team of engaged and proactive administrative colleagues and specialised nursing staff are required for this system to function optimally.

### “Incorporating into haematology and oncology clinics”

“Why did the cardiac physiologist cross the road? To provide an echocardiography service in the lymphoma clinic, of course!” Most cancer patients do not require a cardio-oncologist review, typically needing only echocardiographic monitoring and, occasionally, biomarker monitoring. The ESC cardio-oncology guidelines recommend baseline echocardiograms before certain cancer treatments, which must be arranged promptly to avoid delays in therapy initiation.

The UCLH lymphoma service, as part of its own “one stop shop” approach, brings newly diagnosed patients in for a same-day, comprehensive work-up. This includes clinical assessments, blood tests, radiological imaging, and, if necessary, invasive procedures like biopsies. This setup created an opportunity to incorporate an allied echocardiography service, particularly since many patients would receive high-dose anthracycline chemotherapy, necessitating a baseline echocardiogram.

By offering a cardio-oncology echo appointment on the same day, patients could undergo their echocardiogram alongside their lymphoma related work up, reducing the number of healthcare visits. Importantly, this allowed haematologists to plan treatment with all necessary information at hand. If a cardiac issue was identified, it could be escalated to the cardio-oncologist early, minimising delays in treatment initiation.

Data collected from online medical records for lymphoma patients who accessed the integrated echocardiography service over a twelve-month period from 2023 to 2024 revealed that 89 patients had their echocardiogram through this pathway (see Table 4). Just over half were male, and the majority had non-Hodgkin’s lymphoma (69%). The average LVEF and GLS were within normal ranges, though diastolic dysfunction was common (20%). Nine patients were referred to the cardio-oncology service based on abnormalities on their baseline echo, and they could be quickly accommodated into a cardio-oncology clinic (10 days).

**Table 4:** Patient demographics; *N* = 89 patients with lymphoma seen through the incorporated echocardiography clinic over 12 months

Demographics	% (*n*)
Age (years) – Median [IQR]	43 [33–60]
Male	55% (49)
**Lymphoma Type**	
Hodgkin’s	31% (28)
Non-Hodgkin’s	69% (61)
**Transthoracic Echo Findings**	
LVEF (%) – Median [IQR]	58 [56–60]
GLS (%) – Median [IQR]	-18 [17.9–20]
RV dysfunction	2% (2)
Diastolic dysfunction	20% (18)
Pulmonary hypertension	0
Moderate or severe valve disease	0
**Echo Timing**	
Days from referral – Median [IQR]	5 [0–11]
**Referral to Cardio-Oncology**	
Yes	10% (9)
Days to be seen from referral – Median [range]	10 [5–26]

Presented as median (IQR), mean ± SD or percentage of cohort (n) as appropriate. IQR = interquartile range, SD = standard deviation, LVEF = left ventricular ejection fraction, GLS = global longitudinal strain, RV = right ventricle

### “Physiologist led clinics” 

Physiologist led clinics have been running in other areas of cardiology for a number of years. For instance, valve clinics have been successfully run by cardiac physiologists across many institutions for surveillance of common valve diseases such as aortic stenosis [14, 15]. This concept empowers the physiologist to not only perform the echo, but also undertake a clinical assessment to screen for the impact the underlying condition is having on the patient (which warrants escalation to supporting cardiologists). We have adopted and optimised this format for patients referred for screening of cardiotoxicity following childhood cancer treatment, also known as “late effects” patients. Our late effects cohort includes survivors younger than 25 years at cancer diagnosis, who are more than 5 years from the completion of cancer therapy and were previously treated with anthracycline chemotherapy and/or radiotherapy with cardiac exposure. This particular patient group typically includes those affected by leukemias and lymphomas who have a very good prognosis. However, whilst they may have survived their cancer, they are at risk of numerous long-term cardiovascular complications, including cardiomyopathy, ischaemic heart disease, and valve disease [16]. Guidelines on cardiac surveillance in this cohort currently suggest a screening echography every 2 years if high risk, or every 5 years if moderate risk. Estimation of risk is typically based on the total dose of anthracycline and chest wall radiotherapy received [17, 18]. 

**Fig. 2:**
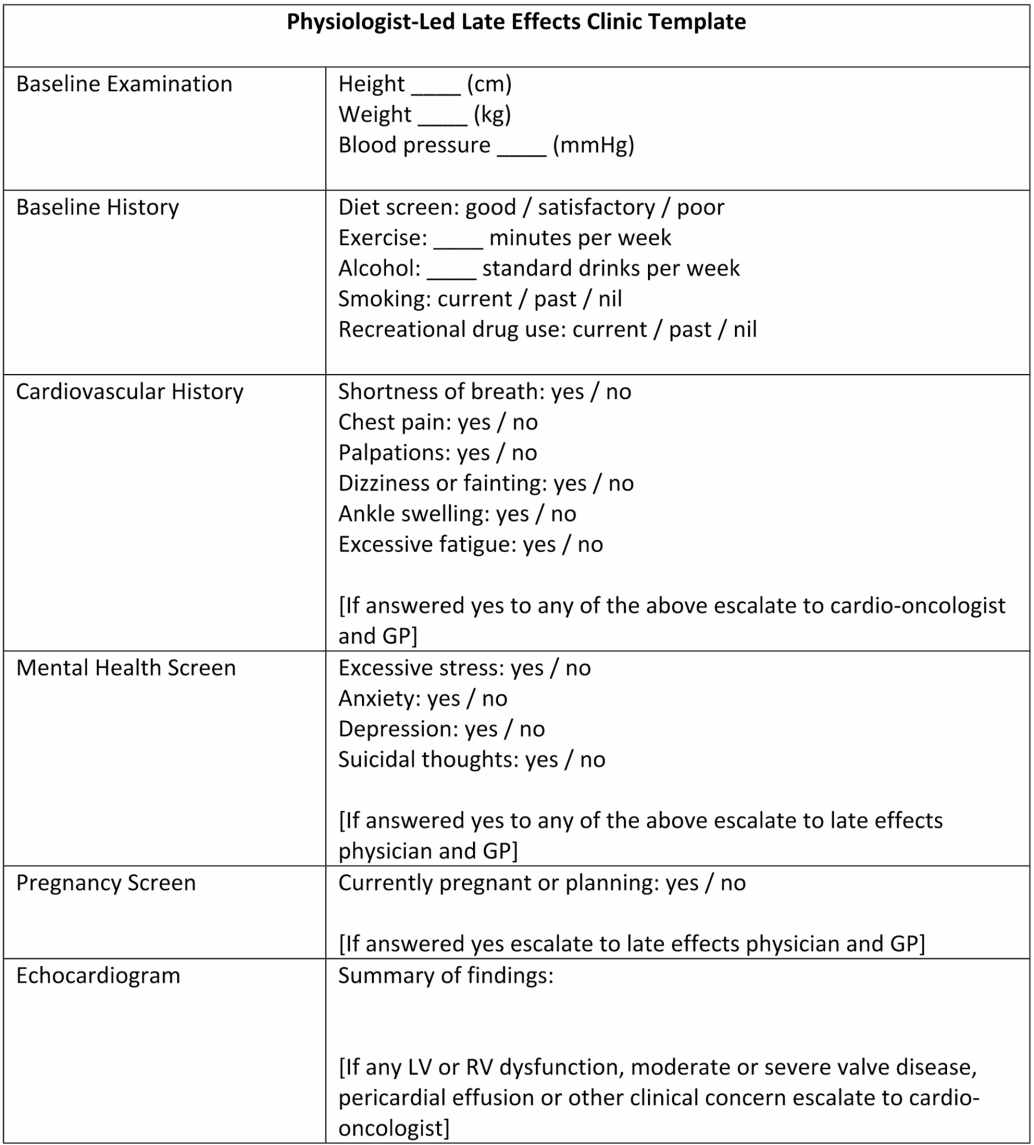
Physiologist led late effects clinic template

**Fig. 3:**
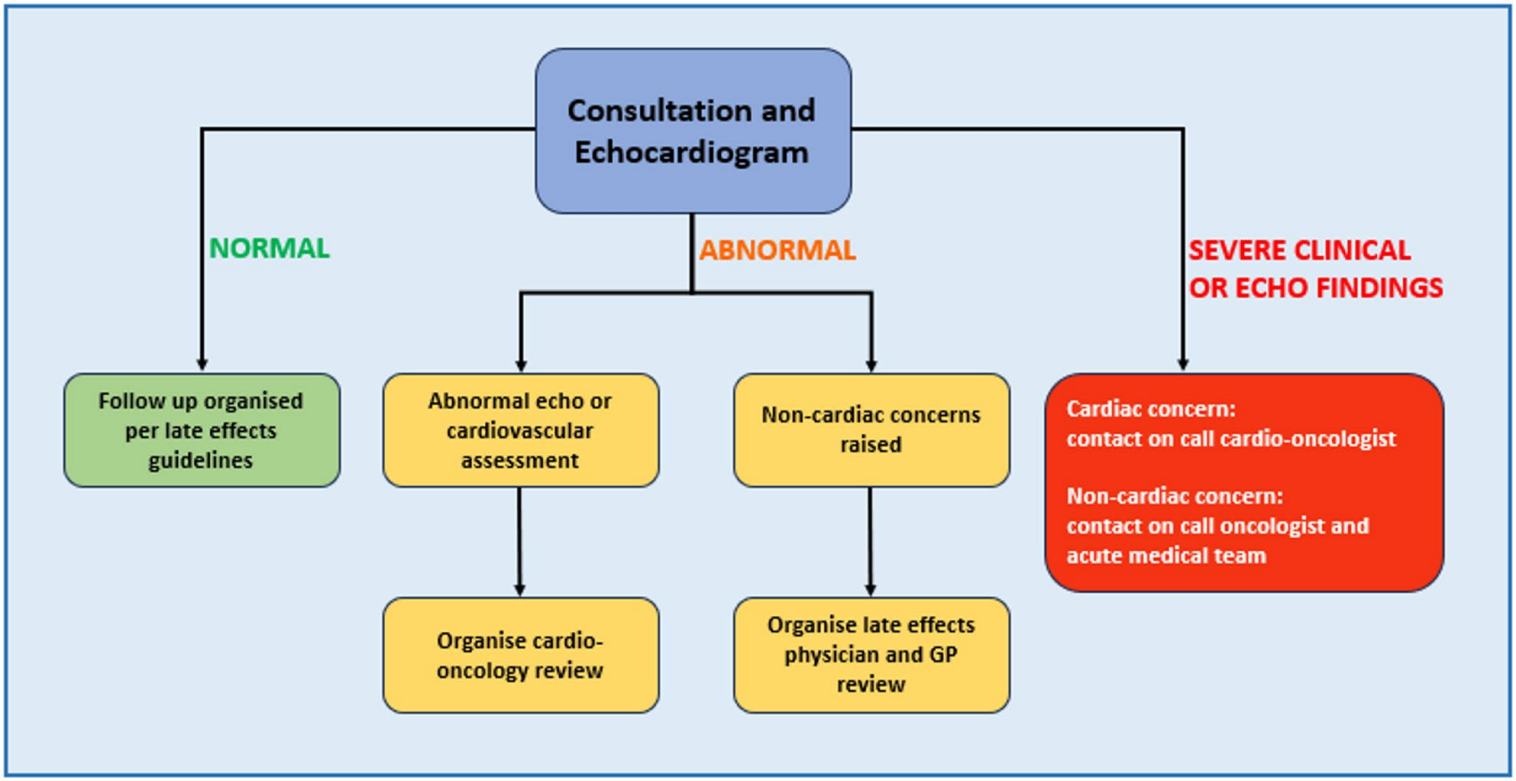
Escalation of findings from the physiologist led late effects clinic

The physiologist led late effects service will see medium and high risk patients based on prior anthracycline dose, being triaged into the clinic if they have no other major morbidity. A physiologist performs an echocardiogram, along with a basic clinical assessment which includes questions screening for cardiac symptoms (Fig. 2). If there is an abnormal echocardiographic finding or clinical assessment, then concerns would immediately be escalated to the cardio-oncologist on call (Fig. 3). Non cardiac clinical concerns are escalated to the late effects specialist consultant, who may be an oncologist or haematologist. The physiologist then completes a letter and appropriate electronic documentation.

From 2023 to 2024 a total of 52 cancer survivors were enrolled in this service. The mean was 29 years (range 19–52), with a balanced gender distribution (27 males, 24 females). The most common cancer types were acute lymphoblastic leukaemia (*n* = 16), Hodgkin lymphoma (*n* = 8), and osteosarcoma (*n* = 5). Of these, 18 (35%) presented with symptoms or findings that warranted further clinical attention. This included 4 patients with borderline or mildly reduced LV systolic function (EF 50–54%), referred for cardiac MRI and had their follow-up intervals shortened for closer monitoring, one was directly referred for cardiology review. Other indications for investigation included palpitations, prolonged QTc interval, and chest pain. Investigations arranged included Holter monitoring, cardiac MRI, CTCA, and biomarker testing, depending on clinical context. A few patients were also referred to Haematology services for review in the context of anaemia, unexplained breathlessness, and significant weight loss. Two patients were referred for psychological counselling.

Organising the late effects service this way has multiple benefits; it reduces hospital visits for cancer survivors, increases capacity for the late effects team to focus on more complex survivors, and importantly offers a unique experience that champions the skillset of our physiologists, who on an organisation survey all report a greater job satisfaction from the expanded role.

#### How did we do it?

#### Protocol development

Standardised protocols were developed to guide the use of echocardiography in cardio-oncology patients through multi-disciplinary meetings involving interested parties. In many cases protocol development was a dynamic process, and procedures were reassessed at future meetings and amended as appropriate. Structure was emulated where possible, such as previously described physiologist led valve clinics. Protocols were ultimately formalised within the hospital network as standard operating procedures (SOPs).

#### Infrastructure and equipment

Setting up the service required investment in advanced echocardiography equipment capable of performing a range of imaging modalities, including 3D echocardiography, strain imaging, and contrast echocardiography. Alongside appropriate equipment and software, dedicated hospital clinics and facilities were also required. Incorporating this into established cancer clinics, required negotiation with providers to procure a suitable clinical space and time so that a safe and efficient service could be provided for patients. Multi-specialty support from cardiology, haematology, and oncology teams was essential to the endeavour.

#### Training and education

A significant aspect of setting up the service was the training and education of staff. This required engaged staff to teach and mentor the physiologists. They received specialised training in recognising cardiotoxicity, as well as support with advanced techniques such as 3D/4D, strain analysis and contrast echocardiography. Continuous professional development programs ensured that all team members remained up to date on the latest advancements in cardio-oncology.

#### Quality control

Quality control is assured through regular departmental audits of provided services. Feedback is also sort from oncology and haematology teams and combined departmental meetings. Cardio-oncologists will oversee echocardiography images during consultation, and interesting or uncertain findings will be discussed at the weekly “echo meetings” or at the cardio-oncology multidisciplinary team (MDT) meeting.

##### Future directions

The field of cardio-oncology is rapidly evolving as new drugs are being discovered, diagnostics and surveillance is improved, and patients are living longer. In particular, the expansion in the use of immunotherapy and CAR T-cell therapy is of particular significance in our patient population at UCLH [19, 20]. Such developments will continue to offer exciting opportunities for cardio-oncologists and cardiac physiologists alike. With engaged physiologists, and productive relationships with haematology and oncology, there will be plenty of opportunities for service optimisation and enhancement. Cardio-oncology echocardiography services will need to remain adaptable and flexible to meet the rising demand for high quality and frequent imaging. Broader training for echocardiographers across the country specific to cardio-oncology may help foster centres of excellence and streamline patients into physiologist led clinics. With a greater understanding of biomarker relevance, these will likely become a more important contribution to patient work up and triage. Collaboration between UK centres and the wider international community will be fundamental to further advance the field and build on the evidence base. This presents a unique opportunity for cardiac physiologists to help improve the outcomes of cancer patients and contribute to shaping the future of cardio-oncology service delivery in the UK.

## Supplementary Information

Below is the link to the electronic supplementary material.

Supplementary Material 1

## Data Availability

Data is provided within the manuscript. Human Ethics and Consent to Participate declarations undertaken where required.
