# Supplementary material for: Echocardiography in cardio-oncology: optimising service delivery
Source: Echo Res Pract. 2026 Apr 20;13:11. doi: 10.1186/s44156-026-00115-5 (PMC13094049; doi:10.1186/s44156-026-00115-5)
Supplement: Supplementary file 1 — Supplementary Material 1 [file 44156_2026_115_MOESM1_ESM.docx]

Standard operating Procedure for Cardio-oncology Transthoracic Echocardiography

*(taken from University College Hospital protocol)*

1. Transthoracic assessment of adult patients before, during and after cancer therapy

1.1 Baseline study:

Standard transthoracic echocardiography is performed.

Emphasis of the following measurements to be noted in the report:

- **Left ventricular systolic function by ejection fraction:**
  - 3D imaging acquisition for LVEF (preferred if possible) or 2D Simpsons method
  - Use of contrast if needed
  - Choose *one optimal method* and use this method for follow up studies
- **Left ventricular longitudinal systolic function:**
  - **2D strain imaging acquisition**
    - Apical three, four, and two-chamber views
    - Images obtained simultaneously (multi-D triplane) maintaining the same 2D frame rate and imaging depth; frame rate between 40 and 90 frames/second
    - Display the segmental strain curves from apical views in a quad
    - Display the global strain in a bull’s-eye plot
  - **TDI S` Septal and Lateral**
- **Pulmonary Artery Systolic Pressure (PASP)**
- **Right Ventricular systolic function**
  - TAPSE
  - TDI S`
  - Take focused RV image in apical 4C view for post-hoc longitudinal strain assessment if needed

1.2 Focused TTE / Follow up study:

Focused studies should only be performed under the following circumstances:

- Full study within the preceding 6 to 12 weeks
- Specific single pathology assessment required (e.g. LVEF / pericardial effusion)
- Pre-agreed with referring clinician

Reporting:

- For focused studies, only the conclusion section needs to be completed
- The study should clearly state it is a limited / focused study, and list the focus of the study
- No other findings need to be reported (and should refer to the original complete study)
- Where a new or unexpected finding is noted, the study should be done in full as per the standard minimum dataset

1.3 Suboptimal Image Quality:

Underestimation of volumes may occur when the endocardium is not adequately visualised. Endocardial border dropout can frequently occur in patients undergoing chemotherapy (e.g. patients with breast cancer after mastectomy and chest irradiation)

The use of myocardial contrast agents could potentially be useful in chemotherapy patients when endocardial dropout occurs. According to current recommendations, contrast should be used when two contiguous LV segments are not well visualised on apical images.

What do we need to perform this study?

- Experienced cardiac physiologist or cardiology resident to cannulate
- Cardiology resident to inject the contrast and complete consent form
- Set up contrast protocol on the machine

Note: Contrast agents are not recommended in conjunction with 3DE or Strain imaging.

Follow the standard operating procedure for LVO Contrast.
